# Supplementary material for: A high throughput bispecific antibody discovery pipeline
Source: Commun Biol. 2023 Apr 7;6:380. doi: 10.1038/s42003-023-04746-w (PMC10082157; doi:10.1038/s42003-023-04746-w)
Supplement: Supplementary file 1 — Supplementary Information [file 42003_2023_4746_MOESM1_ESM.pdf]

SUPPLEMENTARY INFORMATION

A high throughput bispecific antibody discovery pipeline

Author list:
Aude I. Segaliny[a, #], Jayapriya Jayaraman[b, #], Xiaoming Chen[a, $], Jonathan Chong[a, $], Ryan Luxon[a, $], Audrey Fung[a], Qiwei Fu[a], Xianzhi Jiang[a], Rodrigo Rivera[a], Xiaoya Ma[a], Ci Ren[a], Jan Zimak[c], Per Niklas Hedde[b], Yonglei Shang[a], George Wu[a,*] and Weian Zhao[b,c,d,e,f,g,h,*]

Affiliations:
[a] Amberstone Biosciences Inc, Laguna Hills, CA 92653, USA
[b] Department of Biomedical Engineering, University of California, Irvine, Irvine, CA 92697, USA
[c] Department of Pharmaceutical Sciences, University of California, Irvine, Irvine, CA 92697, USA
[d] Sue and Bill Gross Stem Cell Research Center, University of California, Irvine, Irvine, CA 92697, USA
[e] Chao Family Comprehensive Cancer Center, University of California, Irvine, Irvine, CA 92697, USA
[f] Edwards Life Sciences Center for Advanced Cardiovascular Technology, University of California, Irvine, Irvine, CA 92697, USA
[g] Department of Biological Chemistry, University of California, Irvine, Irvine, CA 92697, USA
[h] Institute for Immunology, University of California, Irvine, Irvine, CA 92697, USA

# co-first authors

$ co-second authors

Corresponding authors: George Guikai Wu (george@amberstonebio.com), Weian Zhao (weianz@uci.edu)

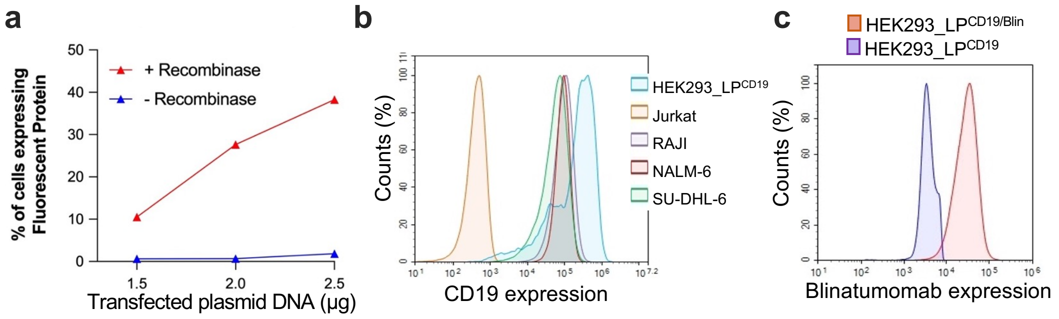

Supplementary Fig. 1. **Establishment of a HEK293 cell line expressing CD19 (target) and a positive CD19xCD3 BiTE (Blinatumomab).** (**a**) Line graph showing the recombinase-dependent integration efficiency at the landing pad of the HEK293 cells, using a mCherry-encoding donor plasmid as a test material. (**b**) Flow cytometry profiling of a selected clone showing moderate to high CD19 ("HEK293_LP^{CD19}"), compared to a few lymphoma lines (RAJI, NALM-6 and SU-DHL-6); Jurkat served as a negative control. (**c**) Flow cytometry validation of HEK293_LP$^{CD19/Blin}$ cells with a CD19xCD3 BiTE (Blinatumomab) that is integrated into the landing pad. The expression of Blinatumomab (6xHis-tagged) was validated using the cell staining with an anti-6xHis antibody.

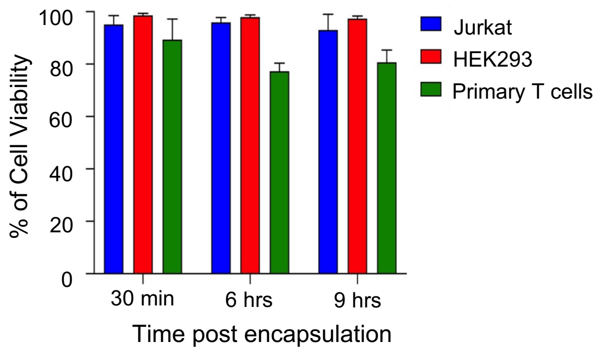

Supplementary Fig. 2. The viability profile of a few human cell types over a duration that is typical of screening assays conducted on single cell discovery platform. Cells are respectively encapsulated in 250-pL droplets and incubated at 37°C under 5% CO2. Droplets are de-emulsified to recover cells in bulk for subsequent 7AAD staining followed by flow cytometry. Jurkat (E6.1), a human leukemia T cell line; HEK293, an immortalized embryonic kidney epithelial cell line; primary T cells: enriched from human PBMCs. Values of mean from $n = 2$ encapsulation experiments are presented as bar graphs with $mean \pm SD$ error bars.

a

Droplets Scanned	Positive Droplets detected	True Positive Rate	Detected Positive Rate	Detection Efficiency
339,800	8702	2.85%	2.56%	89.8%

b

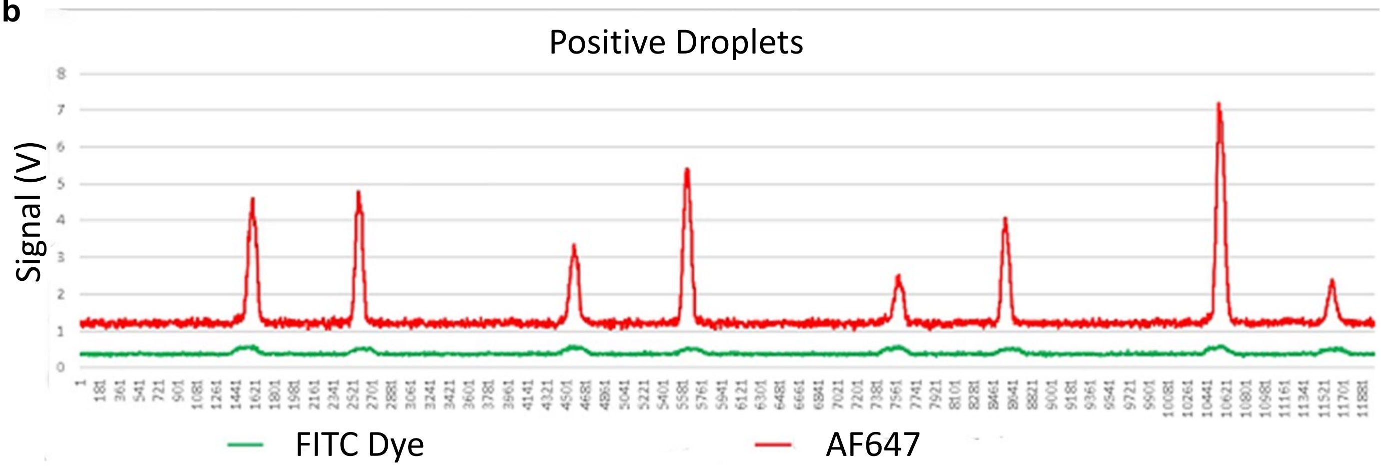

c

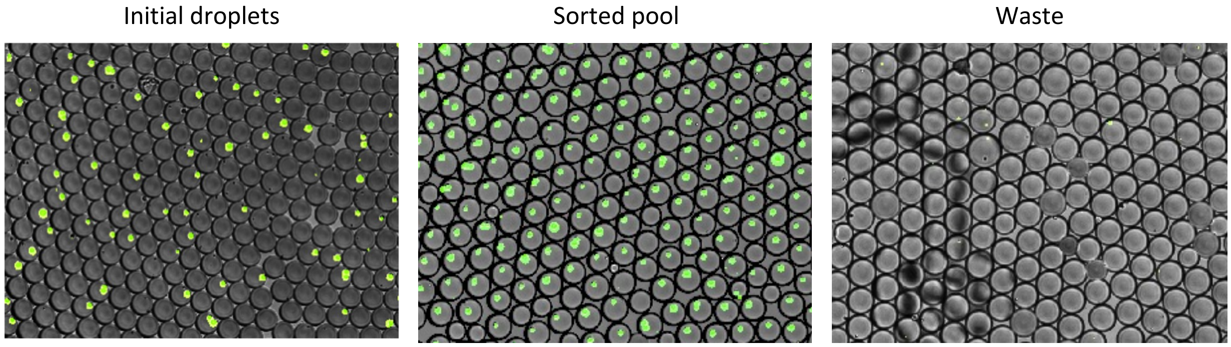

Supplementary Fig. 3. (a) Detection efficiency of our single cell discovery platform. The detection efficiency for intra-droplet fluorescent particles is approx. 90%. This performance metric is determined by an experiment conducted using FITC dyed droplets allowing the detection of empty droplets, spiked with FC AF647 category 3 calibration beads to simulate cells. Beads are spiked into droplets to achieve a calculated positive rate of 3% (estimated percentage of droplets containing beads). Microscope imaging was then used to verify the actual positive rate to be 2.85%. The system then scanned 339,800 droplets and detected 8702 positive droplets using a threshold set at 3 times the Signal-to-Noise (SNR), which is a positive rate of 2.56%. We then divided the system's detected positive rate with the true positive rate to estimate the detection efficiency (89.8%). This experiment was triplicated (n = 3). **(b) Representative PMT detection of positive droplets**. **(c) Microscopic images for droplets before sorting** (*left*), sorted pool(*center*), and waste pool(*right*). Green, GFP reporter signal in activated individual reporter T cells (Jurkat) inside droplets (bright field image). Scale bar, 200 µm.

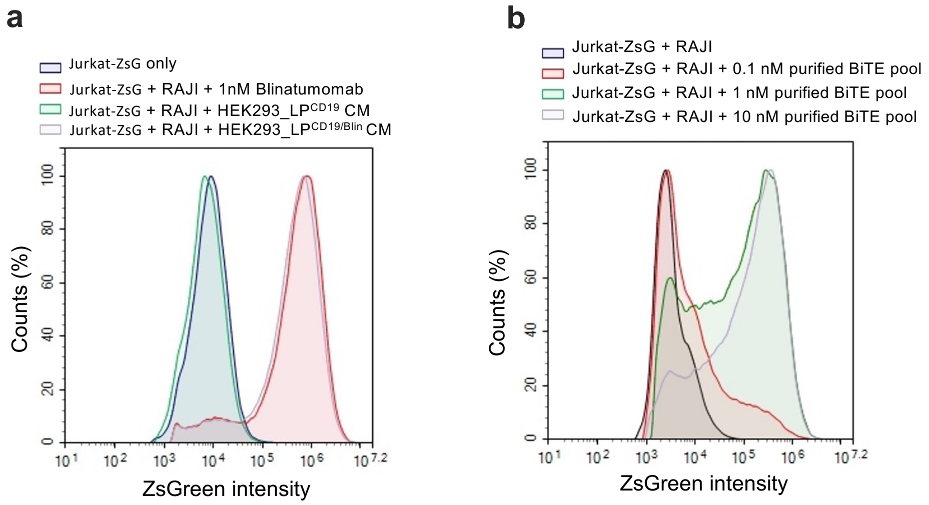

Supplementary Fig. 4. Functional validation of secretion of Blinatumomab and BiTE library by assay of cell culture supernatants. (a) Blinatumomab containing cell culture supernatants (CM) mixed with a co-culture of Jurkat-ZsG and Raji cells for 24 hrs activated Jurkat-ZsG reporter cells. Media containing 1nM recombinant Blinatumomab is used as positive control. (**b**) Jurkat-ZsG reporter cells were co-cultured with HEK293^{CD19} for 24 hours in the presence of purified BiTE pool (0.1, 1 and 10 nM respectively) from the day-3 condition medium of the library cell culture. Reporter activation was measured using flow cytometry.

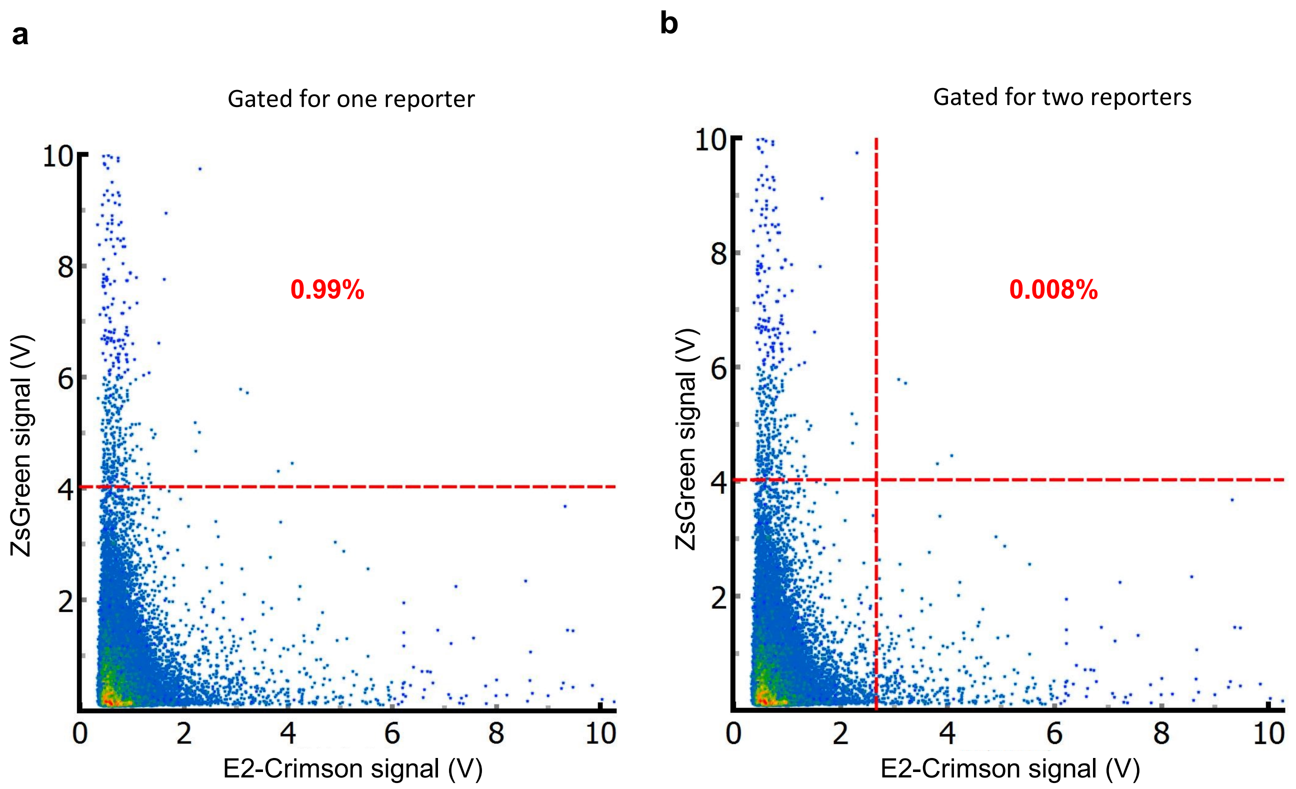

Supplementary Fig. 5. Representation of false positivity rate from detection modules of the single cell discovery platform (a) False positive rate when only ZsGreen reporter signal is used. (b) False positive rate when both ZsGreen and E2-Crimson signal are used.

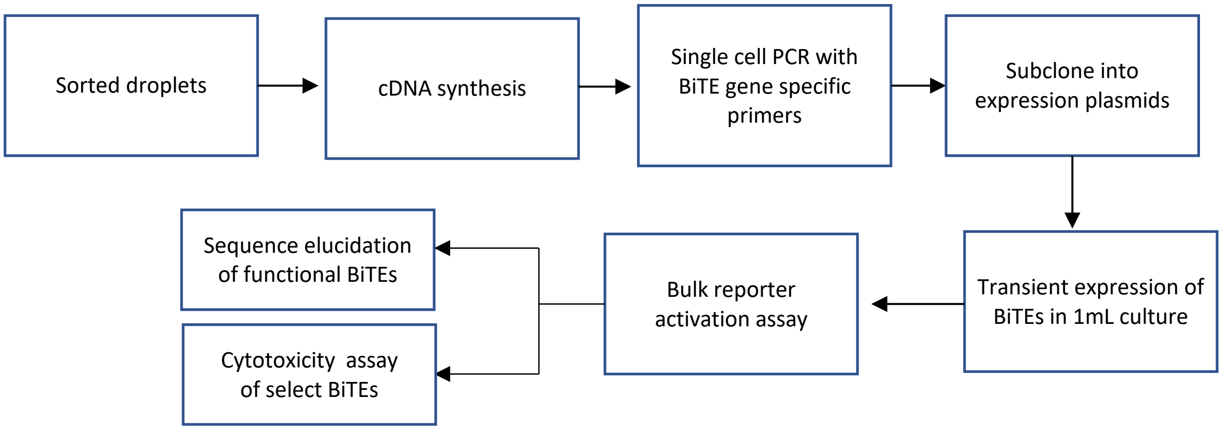

Supplementary Fig. 6. Flowchart illustrating downstream processes after sorting.

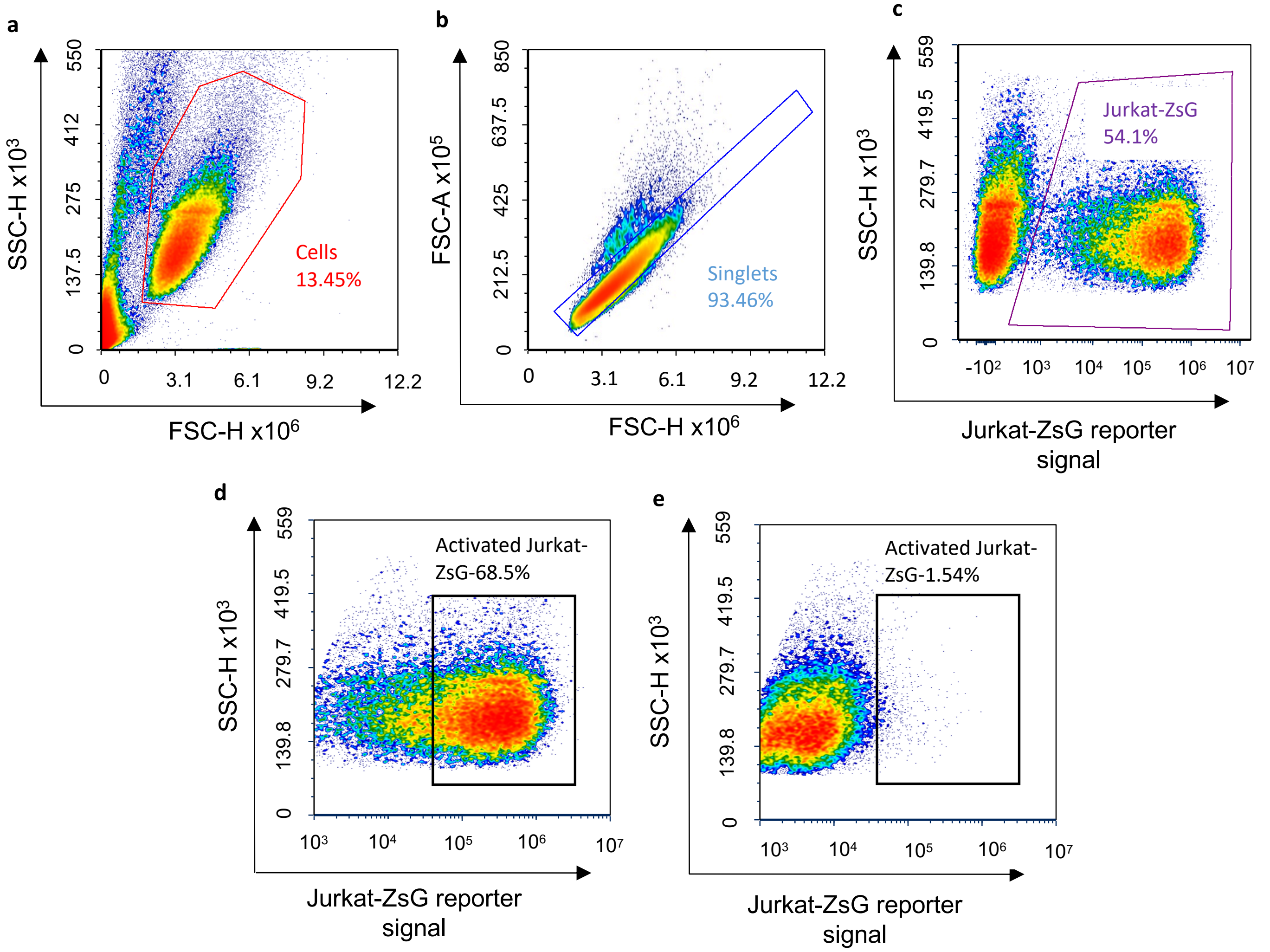

Supplementary Fig. 7. Flow cytometry gating strategy for bulk reporter activation assay. BiTE clones were expressed in Expi293F^{TM} cells and conditioned media from Day 3 expression was used in a coculture of Raji and Jurkat ZsG cells. 24 hrs after co-culture, ZsGreen protein expression from Jurkat-ZsG cells was analyzed by flow cytometry. Representative gating strategy for identifying population of activated Jurkat-ZsG cells is shown for the case where Raji and Jurkat-ZsG cells were co-incubated with Blinatumomab conditioned medium.**(a)** Cell debri was first excluded based on FSC-H/SSC-H gating. **(b)** *S*inglets were isolated with FSC-H/FSC-A gates*.* **(c)** Raji cells were then excluded based on basal ZsG expression from Jurkat-ZsG reporter cells. **(d)** Percentage of activated reporter cells *(bottom left)* were identified by gating for Jurkat-ZsG cells expressing ZsGreen over basal level. **(e)** A negative control consisting of Jurkat-ZsG and Raji cells co-incubated with media from mock transfected Expi293F^{TM} cell line was used to identify basal Jurkat-ZsG fluorescence.

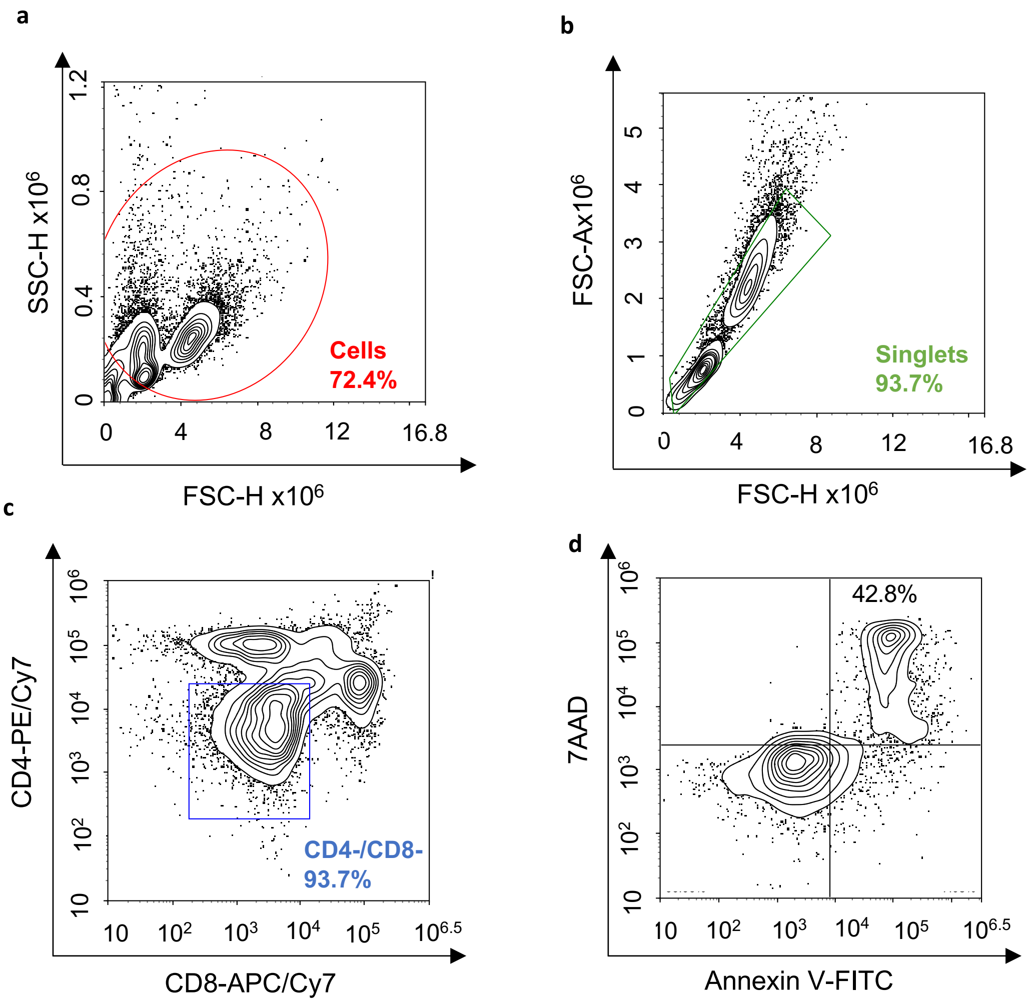

Supplementary Fig. 8. **Flow cytometry gating strategy for in-vitro cytotoxicity assay**. PanT cells isolated from donor PBMCs were co-incubated with Raji cells in presence of purified BiTE clones at different concentrations. 48 hrs after co-culture, cytotoxicity was assessed by flow cytometry. Gating strategy for the flow cytometric analysis is shown for a representative case where panT cells and Raji cells are co-incubated with recombinant Blinatumomab(used as positive control at 0.125 nM). **(a)** Cell debri was excluded with FSC-H/SSC-H gating. **(b)** Singlets were identified using gates for FSC-A/FSC-H. **(c)** Raji cells were identified as a non-T cell population(double negative CD4-CD8- population). **(d)** Annexin V+ and 7AAD+ cells were identified from Raji cell population *(top right quadrant).*

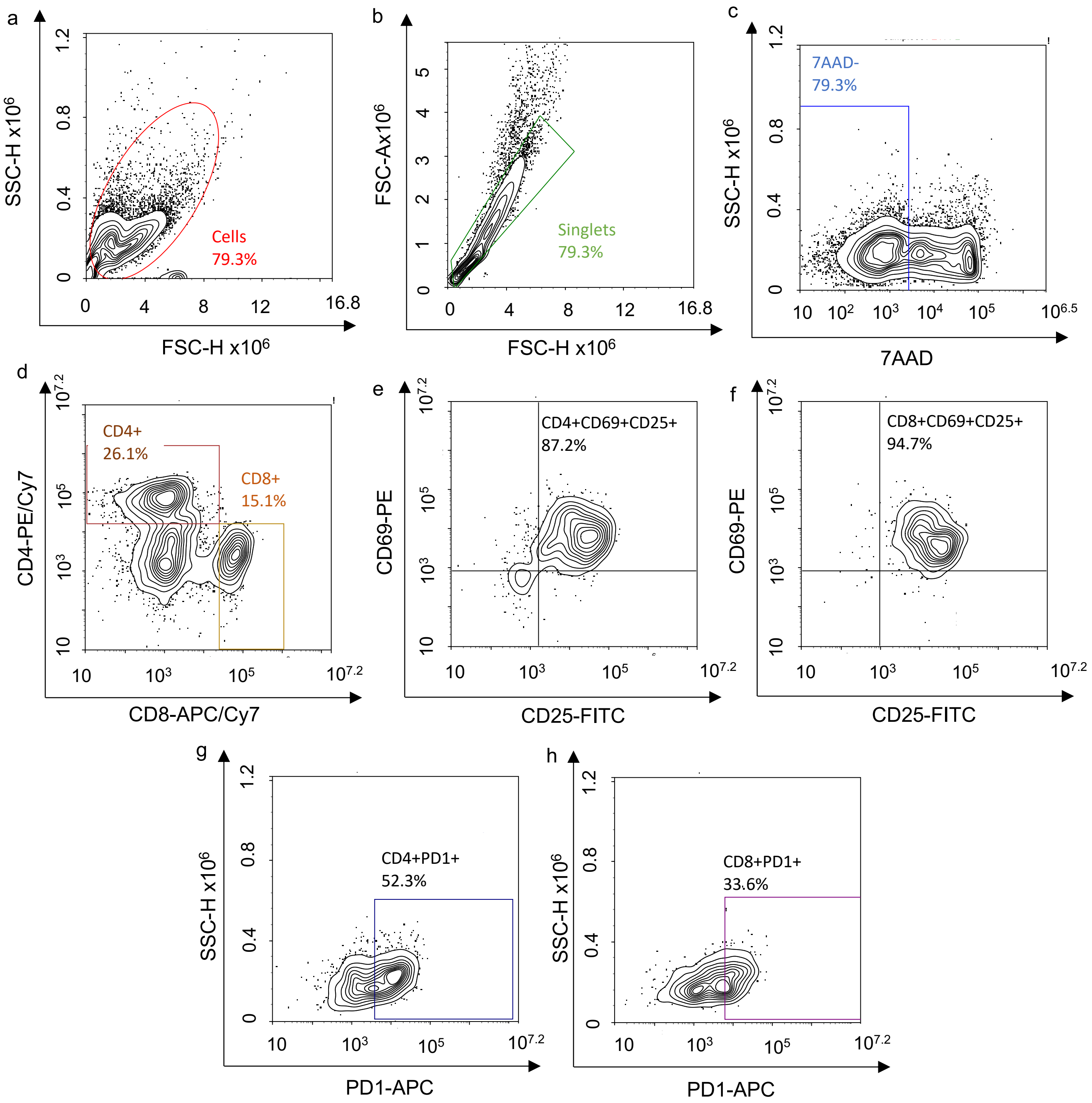

Supplementary Fig. 9. **Flow cytometry gating strategy for in-vitro cytotoxicity assay**. PanT cells isolated from donor PBMCs were isolated from co-incubated with Raji cells in presence of purified BiTE clones at different concentrations. 48 hrs after co-culture, activation was assessed by flow cytometry. Gating strategy for the flow cytometric analysis is shown for a representative case where panT cells and Raji cells are co-incubated with recombinant Blinatumomab(used as positive control at 0.125 nM). **(a)** Cell debri was excluded with FSC-H/SSC-H gating. **(b)** Singlets were identified using gates for FSC-A/FSC-H. **(c)** 7AAD- population was used to gate for live cells **(d)** CD4+ and CD8+ cells were identified. **(e)** Percentage of CD4+CD69+CD25+ cells were identified by subgating for CD69+ and CD25+ from the CD4+ population**. (f)** Percentage of CD8+CD69+CD25+ cells were identified by subgating for CD69+ and CD25+ from the CD8+ population **(g)** Percentage of CD4+PD1+ population was obtained by subgating for PD1+ from the CD4+ population (h) Percentage of CD8+PD1+ population was obtained by subgating for PD1+ from the CD8+ population.

aCD19 scFv	Affinity (nM)	Epitope of binding	CDRH3 length	CDRH3-NNW randomized sequences/scFv	VH/VL orientation
4G7	8.4 nM	Conformationally similar epitope with FMC63, partially overlapping yet distinct from B43.	12	216	2
FMC63	0.4 nM	Discontinuous epitope partially overlapping with B43.	12	216	2
Juno 241	NA	NA	16	288	2
B43	1.5 nM	Loop 216-224, Loop 155-166 on CD19	15	270	1
Total Number of aCD19 variants					1710

Supplementary Table 1. aCD19 scFvs and their characteristics such as affinity, epitope and CDRH3 lengths. The diversity generated in CD19 scFvs by performing site scanning mutagenesis on CDRH3 residues and altering relative orientation of VH-VL regions is also shown.

<table>
<tr><th>aCD3 scFv</th><th>Affinity (nM)</th><th>Epitope of binding</th><th>CDRH3/ Randomization</th><th>VH/VL orientation</th></tr>
<tr><td>OKT3</td><td>0.5 nM</td><td>Discontinuous epitope on CD3ε, middle of extracellular domain near a charged patch</td><td rowspan="5">No randomization of CDRH3 residues</td><td>2</td></tr>
<tr><td>L2K</td><td>110 nM</td><td>Overlapping epitope with OKT3</td><td>2</td></tr>
<tr><td>TRX4</td><td>0.09 nM</td><td>NA</td><td>2</td></tr>
<tr><td>h38E4.v1</td><td>1 nM</td><td>N-terminal portion of CD3ε extracellular domain</td><td>1</td></tr>
<tr><td>Total Number of aCD3 variants</td><td></td><td></td><td>7</td></tr>
</table>

Supplementary Table 2. **aCD3 scFvs and their characteristics such as affinity, epitope of binding**. Diversity in aCD3 scFvs was generated by altering relative orientation of VH-VL regions.

scFv connecting linker	Sequence	Characteristics
Linker 1	GGGGS	Flexible, uncharged, short
Linker 2	GGGGSGGS	Flexible uncharged, long
Linker 3	AEAAAKA	Rigid, charged, short
Linker 4	AEAAAKEAAAKA	Rigid, charged, long

Supplementary Table 3. scFv interconnecting linkers chosen for library generation.
Linkers were varied based on flexibility, charge and length.

**Theoretical library diversity**			
Total number of aCD19 variants	Total number of aCD3 variants	Number of Linkers	Theoretical number of library variants
1,710	7	4	47,880

Supplementary Table 4. Theoretical library diversity of BiTE library calculated as product of number of aCD19 variants, number of aCD3 variants and number of linkers.

	4G7	B43	FMC63	JUNO241
OKT3	9	9	2	8
L2K	15	7	2	10
TRX4	9	8	4	10
h38E4.v1	2	3	0	1

Supplementary Table 5: Distribution of CD19 and CD3 scFv functional pairs in the sorted candidates

scFv sublibrary sequences	Orientation	Amino acid sequence	Target
OKT3-VH-Linker-VL	VH-VL	QVQLQQSGAELARPGASVKMSCKASGYTFTRYTMHWVKQRPGQGLEWIG<br>YINPSRGYTNYNQKFKDKATLTTDKSSSTAYMQLSSLTSEDSAVYYCAR<br>YYDDHYCLDYWGQGTTVTVSSVEGGSGGSGGSGGSGGVDDIQLTQSPAI<br>MSASPGEKVTMTCSASSSVSYMNWYQQKSGTSPKRWIYDTSKLASGVPA<br>HFRGSGSGTSYSLTISGMEAEDAATYYCQQWSSNPFTFGSGTKLEIK	CD3 scFvs
L2K-VH-linker-VL	VH-VL	QVQLQQSGAELARPGASVKMSCKTSGYTFTRYTMHWVKQRPGQGLEWIG<br>YINPSRGYTNYNQKFKDKATLTTDKSSSTAYMQLSSLTSEDSAVYYCAR<br>YYDDHYCLDYWGQGTTVTVSSVEGGSGGSGGSGGSGGVDDIQLTQSPAI<br>MSASPGEKVTMTCRASSSVSYMNWYQQKSGTSPKRWIYDTSKVASGVPY<br>RFSGSGSGTSYSLTISSMEAEDAATYYCQQWSSNPLTFGAGTKLEIK	
hu38E4.v1_VH-linker-VL	VH-VL	QVQLQQSGAEVKKPGASVKVSCKASGFTFTSYYIHWVRQAPGQGLEWIG<br>WIYPENDNTKYNEKFKDRVTITADTSTSTAYLELSSLRSEDTAVYYCAR<br>DGYSRYYFDYWGQGTTVTVSSVEGGSGGSGGSGGSGGVDDIQLTQSPDS<br>LAVSLGERATINCKSSQSLLNSRTRKNYLAWYQQKPGQSPKLLIYWTST<br>RKSGVPDRFSGSGSGTDFTLTISSLQAEDVAVYYCKQSFILRTFGQGTK<br>LEIK	
TRX4_VH-Linker-VL	VH-VL	QVQLQQGGGLVQPGGSLRLSCAASGFTFSSFPMAWVRQAPGKGLEWVST<br>ISTSGGRTYYRDSVKGRFTISRDNSKNTLYLQMNSLRAEDTAVYYCAKF<br>RQYSGGFDYWGQGTTVTVSSVEGGSGGSGGSGGSGGVDDIQLTQPNSVS<br>TSLGSTVKLSCTLSSGNIENNYVHWYQLYEGRSPTTMIYDDDKRPDGVP<br>DRFSGSIDRSSNSAFLTIHNVAIEDEAIYFCHSYVSSFNVFGGGTKLEI<br>K	
OKT3-VL-Linker-VH	VL-VH	DIQLTQSPAIMSASPGEKVTMTCSASSSVSYMNWYQQKSGTSPKRWIYD<br>TSKLASGVPAHFRGSGSGTSYSLTISGMEAEDAATYYCQQWSSNPFTFG<br>SGTKLEIKVEGGSGGSGGSGGSGGVDQVQLQQSGAELARPGASVKMSCK<br>ASGYTFTRYTMHWVKQRPGQGLEWIGYINPSRGYTNYNQKFKDKATLTT<br>DKSSSTAYMQLSSLTSEDSAVYYCARYYDDHYCLDYWGQGTTVTVSS	

hu38E4.v1_VL-linker-VH	VL-VH	DIQLTQSPDSLAVSLGERATINCKSSQSLLNSRTRKNYLAWYQQKPGQSPKLLIYWTSTRKSGVPDRFSGSGSGTDFTLTISSLQAEDVAVYYCKQSFILRTFGQGTKLEIKVEGGSGGSGGSGGSGGVDQVQLQQSGAEVKKPGASVKVSCKASGFTFTSYYIHWVRQAPGQGLEWIGWIYPENDNTKYNEKFKDRVTITADTSTSTAYLELSSLRSEDTAVYYCARDGYSRYYFDYWGQGTTVTVSS	CD3 scFvs
TRX4-VL-Linker-VH	VL-VH	DIQLTQPNSVSTSLGSTVKLSCTLSSGNIENNYVHWYQLYEGRSPTTMIYDDDKRPDGVPDRFSGSIDRSSNSAFLTIHNVAIEDEAIYFCHSYVSSFNVFGGGTKLEIKVEGGSGGSGGSGGSGGVDQVQLQQGGGLVQPGGSLRLSCAASGFTFSSFPMAWVRQAPGKGLEWVSTISTSGGRTYYRDSVKGRFTISRDNSKNTLYLQMNSLRAEDTAVYYCAKFRQYSGGFDYWGQGTTVTVSS	
Juno-241-VH-linker-VL	VH-VL	QVQLQQSGGGLVQPGRSLRLSCAASGFTFDDYAMHWVRQAPGKGLEWVSGISWNSGSIGYADSVKGRFTISRDNAKNSLYLQMNSLRAEDTAVYYCARDQGYHYYDSAEHAFDIWGQGTTVTVSSGGGGSGGGGSGGGGSDIQLTQSPSSLSASVGDRVTVTCQASQDISNYLNWYQQKPGRAPKLLIYDASNVKAGVPSRFSGGGSGTDFTLTISSLQPEDFATYYCQQSYSTPQAYTFGQGTKLEIK	CD19 scFvs
4G7-VH-linker-VL	VH-VL	QVQLQQSGPELIKPGASVKMSCKASGYTFTSYVMHWVKQKPGQGLEWIGYINPYNDGTKYNEKFKGKATLTSDKSSSTAYMELSSLTSEDSAVYYCARGTYYYGSRVFDYWGQGTTVTVSSGGGGSGGGGSGGGGSDIQLTQAAPSIPVTPGESVSISCRSSKSLLNSNGNTYLYWFLQRPGQSPQLLIYRMSNLASGVPDRFSGSGSGTAFTLRISRVEAEDVGVYYCMQHLEYPFTFGAGTKLEIK	
FMC63-VH-Linker-VL	VH-VL	QVQLQQSGPGLVAPSQSLSVTCTVSGVSLPDYGVSWIRQPPRKGLEWLGVIWGSETTYYNSALKSRLTIIKDNSKSQVFLKMNSLQTDDTAIYYCAKHYYYGGSYAMDYWGQGTTVTVSSGGGGSGGGGSGGGGSDIQLTQTTSSLSASLGDRVTISCRASQDISKYLNWYQQKPDGTVKLLIYHTSRLHSGVPSRFSGSGSGTDYSLTISNLEQEDIATYFCQQGNTLPYTFGGGTKLEIK	

B43-CD19-VL-linker-VH	VL-VH	DIQLTQSPASLAVSLGQRATISCKASQSVDYDGDSYLNWYQQIPGQPPK<br>LLIYDASNLVSGIPPRFSGSGSGTDFTLNIHPVEKVDAATYHCQQSTED<br>PWTFGGGTKLEIKGGGGSGGGGSGGGGSQVQLQQSGAELVRPGSSVKIS<br>CKASGYAFSSYWMNWVKQRPGQGLEWIGQIWPGDGDTNYNGKFKGKATL<br>TADESSSTAYMQLSSLASEDSAVYFCARRETTTVGRYYYAMDYWGQGTT<br>VTVSS	CD19 scFvs
Juno-241-VL-linker-VH	VL-VH	DIQLTQSPSSLSASVGDRVTVTCQASQDISNYLNWYQQKPGRAPKLLIY<br>DASNVKAGVPSRFSGGGSGTDFTLTISSLQPEDFATYYCQQSYSTPQAY<br>TFGQGTKLEIKGGGGSGGGGSGGGGSQVQLQQSGGGLVQPGRSLRLSCA<br>ASGFTFDDYAMHWVRQAPGKGLEWVSGISWNSGSIGYADSVKGRFTISR<br>DNAKNSLYLQMNSLRAEDTAVYYCARDQGYHYYDSAEHAFDIWGQGTTV<br>TVSS	
4G7-VL-linker-VH	VL-VH	DIQLTQAAPSIPVTPGESVSISCRSSKSLLNSNGNTYLYWFLQRPGQSP<br>QLLIYRMSNLASGVPDRFSGSGSGTAFTLRISRVEAEDVGVYYCMQHLE<br>YPFTFGAGTKLEIKGGGGSGGGGSGGGGSQVQLQQSGPELIKPGASVKM<br>SCKASGYTFTSYVMHWVKQKPGQGLEWIGYINPYNDGTKYNEKFKGKAT<br>LTSDKSSSTAYMELSSLTSEDSAVYYCARGTYYYGSRVFDYWGQGTTVT<br>VSS	
FMC63-VL-linker VH	VL-VH	DIQLTQTTSSLSASLGDRVTISCRASQDISKYLNWYQQKPDGTVKLLIY<br>HTSRLHSGVPSRFSGSGSGTDYSLTISNLEQEDIATYFCQQGNTLPYTF<br>GGGTKLEIKGGGGSGGGGSGGGGSQVQLQQSGPGLVAPSQSLSVTCTVS<br>GVSLPDYGVSWIRQPPRKGLEWLGVIWGSETTYYNSALKSRLTIIKDNS<br>KSQVFLKMNSLQTDDTAIYYCAKHYYYGGSYAMDYWGQGTTVTVSS	

Blinatumomab® sequence used in HEK293_LP$^{CD19/Blin}$ cell lines	$CD19_{VL\text{-}VH\text{-}}$ $CD3_{VH\text{-}VL}$	DIQLTQSPASLAVSLGQRATISCKASQSVDYDGDSYLNWYQQIPGQPPK<br>LLIYDASNLVSGIPPRFSGSGSGTDFTLNIHPVEKVDAATYHCQQSTED<br>PWTFGGGTKLEIKGGGGSGGGGSGGGGSQVQLQQSGAELVRPGSSVKIS<br>CKASGYAFSSYWMNWVKQRPGQGLEWIGQIWPGDGDTNYNGKFKGKATL<br>TADESSSTAYMQLSSLASEDSAVYFCARRETTTVGRYYYAMDYWGQGTT<br>VTVSSGGGGSQVQLQQSGAELARPGASVKMSCKTSGYTFTRYTMHWVKQ<br>RPGQGLEWIGYINPSRGYTNYNQKFKDKATLTTDKSSSTAYMQLSSLTS<br>EDSAVYYCARYYDDHYCLDYWGQGTTVTVSSVEGGSGGSGGSGGSGGVD<br>DIQLTQSPAIMSASPGEKVTMTCRASSSVSYMNWYQQKSGTSPKRWIYD<br>TSKVASGVPYRFSGSGSGTSYSLTISSMEAEDAATYYCQQWSSNPLTFG<br>AGTKLEIKHHHHHH	CD19xCD3 BiTE

Supplementary Table 6: Amino acid sequences of CD3 and CD19 scFv sublibraries. N-terminal sequence of all VH domains was normalized to QVQLQQ and C-terminal domains of all VH sequences were normalized to TVTVSS. N-terminal sequence of all VL domains was normalized to DIQLTQ and C-terminal sequence of all VL domains was normalized to TKLEIK. The Blinatumomab sequence used for generation of control cell line HEK293_LP$^{CD19/Blin}$ is also shown.

CDRH3_NNW_Primer	Primer sequence 5'-3'
4G7_VH_NNW_1	CCTGACCTCTGAGGACAGCGCCGTGTACTATTGTGCCAGANNWACCTACTACTACGGCAGCCGGGTGTTCGATTATTGGGGCCAGGGAACCACCG
4G7_VH_NNW_2	CCTGACCTCTGAGGACAGCGCCGTGTACTATTGTGCCAGAGGCNNWTACTACTACGGCAGCCGGGTGTTCGATTATTGGGGCCAGGGAACCACCG
4G7_VH_NNW_3	CCTGACCTCTGAGGACAGCGCCGTGTACTATTGTGCCAGAGGCACCNNWTACTACGGCAGCCGGGTGTTCGATTATTGGGGCCAGGGAACCACCG
4G7_VH_NNW_4	CCTGACCTCTGAGGACAGCGCCGTGTACTATTGTGCCAGAGGCACCTACNNWTACGGCAGCCGGGTGTTCGATTATTGGGGCCAGGGAACCACCG
4G7_VH_NNW_5	CCTGACCTCTGAGGACAGCGCCGTGTACTATTGTGCCAGAGGCACCTACTACNNWGGCAGCCGGGTGTTCGATTATTGGGGCCAGGGAACCACCG
4G7_VH_NNW_6	CCTGACCTCTGAGGACAGCGCCGTGTACTATTGTGCCAGAGGCACCTACTACTACNNWAGCCGGGTGTTCGATTATTGGGGCCAGGGAACCACCG
4G7_VH_NNW_7	CCTGACCTCTGAGGACAGCGCCGTGTACTATTGTGCCAGAGGCACCTACTACTACGGCNNWCGGGTGTTCGATTATTGGGGCCAGGGAACCACCG
4G7_VH_NNW_8	CCTGACCTCTGAGGACAGCGCCGTGTACTATTGTGCCAGAGGCACCTACTACTACGGCAGCNNWGTGTTCGATTATTGGGGCCAGGGAACCACCG
4G7_VH_NNW_9	CCTGACCTCTGAGGACAGCGCCGTGTACTATTGTGCCAGAGGCACCTACTACTACGGCAGCCGGNNWTTCGATTATTGGGGCCAGGGAACCACCG
4G7_VH_NNW_10	CCTGACCTCTGAGGACAGCGCCGTGTACTATTGTGCCAGAGGCACCTACTACTACGGCAGCCGGGTGNNWGATTATTGGGGCCAGGGAACCACCG
4G7_VH_NNW_11	CCTGACCTCTGAGGACAGCGCCGTGTACTATTGTGCCAGAGGCACCTACTACTACGGCAGCCGGGTGTTCNNWTATTGGGGCCAGGGAACCACCG
4G7_VH_NNW_12	CCTGACCTCTGAGGACAGCGCCGTGTACTATTGTGCCAGAGGCACCTACTACTACGGCAGCCGGGTGTTCGATNNWTGGGGCCAGGGAACCACCG
FMC63_VH_NNW_1	CCTGCAGACCGACGACACCGCCATCTACTACTGCGCCAAGNNWTACTACTACGGCGGCAGCTACGCCATGGATTATTGGGGCCAGGGCACCACCGT
FMC63_VH_NNW_2	CCTGCAGACCGACGACACCGCCATCTACTACTGCGCCAAGCACNNWTACTACGGCGGCAGCTACGCCATGGATTATTGGGGCCAGGGCACCACCGT
FMC63_VH_NNW_3	CCTGCAGACCGACGACACCGCCATCTACTACTGCGCCAAGCACTACNNWTACGGCGGCAGCTACGCCATGGATTATTGGGGCCAGGGCACCACCGT
FMC63_VH_NNW_4	CCTGCAGACCGACGACACCGCCATCTACTACTGCGCCAAGCACTACTACNNWGGCGGCAGCTACGCCATGGATTATTGGGGCCAGGGCACCACCGT
FMC63_VH_NNW_5	CCTGCAGACCGACGACACCGCCATCTACTACTGCGCCAAGCACTACTACTACNNWGGCAGCTACGCCATGGATTATTGGGGCCAGGGCACCACCGT
FMC63_VH_NNW_6	CCTGCAGACCGACGACACCGCCATCTACTACTGCGCCAAGCACTACTACTACGGCNNWAGCTACGCCATGGATTATTGGGGCCAGGGCACCACCGT
FMC63_VH_NNW_7	CCTGCAGACCGACGACACCGCCATCTACTACTGCGCCAAGCACTACTACTACGGCGGCNNWTACGCCATGGATTATTGGGGCCAGGGCACCACCGT
FMC63_VH_NNW_8	CCTGCAGACCGACGACACCGCCATCTACTACTGCGCCAAGCACTACTACTACGGCGGCAGCNNWGCCATGGATTATTGGGGCCAGGGCACCACCGT
FMC63_VH_NNW_9	CCTGCAGACCGACGACACCGCCATCTACTACTGCGCCAAGCACTACTACTACGGCGGCAGCTACNNWATGGATTATTGGGGCCAGGGCACCACCGT
FMC63_VH_NNW_10	CCTGCAGACCGACGACACCGCCATCTACTACTGCGCCAAGCACTACTACTACGGCGGCAGCTACGCCNNWGATTATTGGGGCCAGGGCACCACCGT
FMC63_VH_NNW_11	CCTGCAGACCGACGACACCGCCATCTACTACTGCGCCAAGCACTACTACTACGGCGGCAGCTACGCCATGNNWTATTGGGGCCAGGGCACCACCGT
FMC63_VH_NNW_12	CCTGCAGACCGACGACACCGCCATCTACTACTGCGCCAAGCACTACTACTACGGCGGCAGCTACGCCATGGATNNWTGGGGCCAGGGCACCACCGT
Juno241_VH_NNW_1	CCTGAGAGCCGAGGACACCGCCGTGTACTACTGTGCCAGANNWCAGGGCTACCACTACTACGACTCTGCCGAGCACGCCTTCGATATCTGGGGCCAGGGAACCACCGT
Juno241_VH_NNW_2	CCTGAGAGCCGAGGACACCGCCGTGTACTACTGTGCCAGAGATNNWGGCTACCACTACTACGACTCTGCCGAGCACGCCTTCGATATCTGGGGCCAGGGAACCACCGT
Juno241_VH_NNW_3	CCTGAGAGCCGAGGACACCGCCGTGTACTACTGTGCCAGAGATCAGNNWTACCACTACTACGACTCTGCCGAGCACGCCTTCGATATCTGGGGCCAGGGAACCACCGT
Juno241_VH_NNW_4	CCTGAGAGCCGAGGACACCGCCGTGTACTACTGTGCCAGAGATCAGGGCNNWCACTACTACGACTCTGCCGAGCACGCCTTCGATATCTGGGGCCAGGGAACCACCGT
Juno241_VH_NNW_5	CCTGAGAGCCGAGGACACCGCCGTGTACTACTGTGCCAGAGATCAGGGCTACNNWTACTACGACTCTGCCGAGCACGCCTTCGATATCTGGGGCCAGGGAACCACCGT
Juno241_VH_NNW_6	CCTGAGAGCCGAGGACACCGCCGTGTACTACTGTGCCAGAGATCAGGGCTACCACNNWTACGACTCTGCCGAGCACGCCTTCGATATCTGGGGCCAGGGAACCACCGT
Juno241_VH_NNW_7	CCTGAGAGCCGAGGACACCGCCGTGTACTACTGTGCCAGAGATCAGGGCTACCACTACNNWGACTCTGCCGAGCACGCCTTCGATATCTGGGGCCAGGGAACCACCGT
Juno241_VH_NNW_8	CCTGAGAGCCGAGGACACCGCCGTGTACTACTGTGCCAGAGATCAGGGCTACCACTACTACNNWTCTGCCGAGCACGCCTTCGATATCTGGGGCCAGGGAACCACCGT
Juno241_VH_NNW_9	CCTGAGAGCCGAGGACACCGCCGTGTACTACTGTGCCAGAGATCAGGGCTACCACTACTACGACNNWGCCGAGCACGCCTTCGATATCTGGGGCCAGGGAACCACCGT
Juno241_VH_NNW_10	CCTGAGAGCCGAGGACACCGCCGTGTACTACTGTGCCAGAGATCAGGGCTACCACTACTACGACTCTNNWGAGCACGCCTTCGATATCTGGGGCCAGGGAACCACCGT
Juno241_VH_NNW_11	CCTGAGAGCCGAGGACACCGCCGTGTACTACTGTGCCAGAGATCAGGGCTACCACTACTACGACTCTGCCNNWCACGCCTTCGATATCTGGGGCCAGGGAACCACCGT
Juno241_VH_NNW_12	CCTGAGAGCCGAGGACACCGCCGTGTACTACTGTGCCAGAGATCAGGGCTACCACTACTACGACTCTGCCGAGNNWGCCTTCGATATCTGGGGCCAGGGAACCACCGT
Juno241_VH_NNW_13	CCTGAGAGCCGAGGACACCGCCGTGTACTACTGTGCCAGAGATCAGGGCTACCACTACTACGACTCTGCCGAGCACNNWTTCGATATCTGGGGCCAGGGAACCACCGT
Juno241_VH_NNW_14	CCTGAGAGCCGAGGACACCGCCGTGTACTACTGTGCCAGAGATCAGGGCTACCACTACTACGACTCTGCCGAGCACGCCNNWGATATCTGGGGCCAGGGAACCACCGT
Juno241_VH_NNW_15	CCTGAGAGCCGAGGACACCGCCGTGTACTACTGTGCCAGAGATCAGGGCTACCACTACTACGACTCTGCCGAGCACGCCTTCNNWATCTGGGGCCAGGGAACCACCGT
Juno241_VH_NNW_16	CCTGAGAGCCGAGGACACCGCCGTGTACTACTGTGCCAGAGATCAGGGCTACCACTACTACGACTCTGCCGAGCACGCCTTCGATNNWTGGGGCCAGGGAACCACCGT
B43_VH_NNW_1	CCTGGCCAGCGAAGATAGCGCCGTGTACTTCTGTGCCAGANNWGAAACCACCACCGTGGGCAGATATTACTACGCCATGGACTACTGGGGCCAGGGCACCACCGT
B43_VH_NNW_2	CCTGGCCAGCGAAGATAGCGCCGTGTACTTCTGTGCCAGACGGNNWACCACCACCGTGGGCAGATATTACTACGCCATGGACTACTGGGGCCAGGGCACCACCGT

B43_VH_NNW_3	CCTGGCCAGCGAAGATAGCGCCGTGTACTTCTGTGCCAGACGGGAANNWACCACCGTGGGCAGATATTACTACGCCATGGACTACTGGGGCCAGGGCACCACCGT
B43_VH_NNW_4	CCTGGCCAGCGAAGATAGCGCCGTGTACTTCTGTGCCAGACGGGAAACCNNWACCGTGGGCAGATATTACTACGCCATGGACTACTGGGGCCAGGGCACCACCGT
B43_VH_NNW_5	CCTGGCCAGCGAAGATAGCGCCGTGTACTTCTGTGCCAGACGGGAAACCACCNNWGTGGGCAGATATTACTACGCCATGGACTACTGGGGCCAGGGCACCACCGT
B43_VH_NNW_6	CCTGGCCAGCGAAGATAGCGCCGTGTACTTCTGTGCCAGACGGGAAACCACCACCNNWGGCAGATATTACTACGCCATGGACTACTGGGGCCAGGGCACCACCGT
B43_VH_NNW_7	CCTGGCCAGCGAAGATAGCGCCGTGTACTTCTGTGCCAGACGGGAAACCACCACCGTGNNWAGATATTACTACGCCATGGACTACTGGGGCCAGGGCACCACCGT
B43_VH_NNW_8	CCTGGCCAGCGAAGATAGCGCCGTGTACTTCTGTGCCAGACGGGAAACCACCACCGTGGGCNNWTATTACTACGCCATGGACTACTGGGGCCAGGGCACCACCGT
B43_VH_NNW_9	CCTGGCCAGCGAAGATAGCGCCGTGTACTTCTGTGCCAGACGGGAAACCACCACCGTGGGCAGANNWTACTACGCCATGGACTACTGGGGCCAGGGCACCACCGT
B43_VH_NNW_10	CCTGGCCAGCGAAGATAGCGCCGTGTACTTCTGTGCCAGACGGGAAACCACCACCGTGGGCAGATATNNWTACGCCATGGACTACTGGGGCCAGGGCACCACCGT
B43_VH_NNW_11	CCTGGCCAGCGAAGATAGCGCCGTGTACTTCTGTGCCAGACGGGAAACCACCACCGTGGGCAGATATTACNNWGCCATGGACTACTGGGGCCAGGGCACCACCGT
B43_VH_NNW_12	CCTGGCCAGCGAAGATAGCGCCGTGTACTTCTGTGCCAGACGGGAAACCACCACCGTGGGCAGATATTACTACNNWATGGACTACTGGGGCCAGGGCACCACCGT
B43_VH_NNW_13	CCTGGCCAGCGAAGATAGCGCCGTGTACTTCTGTGCCAGACGGGAAACCACCACCGTGGGCAGATATTACTACGCCNNWGACTACTGGGGCCAGGGCACCACCGT
B43_VH_NNW_14	CCTGGCCAGCGAAGATAGCGCCGTGTACTTCTGTGCCAGACGGGAAACCACCACCGTGGGCAGATATTACTACGCCATGNNWTACTGGGGCCAGGGCACCACCGT
B43_VH_NNW_15	CCTGGCCAGCGAAGATAGCGCCGTGTACTTCTGTGCCAGACGGGAAACCACCACCGTGGGCAGATATTACTACGCCATGGACNNWTGGGGCCAGGGCACCACCGT

Supplementary Table 7: Primer sequences encoding NNW codons at each CDRH3 residue of CD19 scFvs

Oligo name	Oligo sequence	Decription
oBiTE_21	AGATCCTCCGCCTCCCTTGATTTCCAGCTTGGT	GGGGS,VL-Cterm-Rev
oBiTE_22	AGATCCACCAGATCCTCCGCCTCCCTTGATTTCCAGCTTGGT	GGGGSGGS,VL-Cterm-Rev
oBiTE_23	AGCTTTAGCAGCGGCTTCAGCCTTGATTTCCAGCTTGGT	AEAAAKA,VL-Cterm-Rev
oBiTE_24	AGCTTTGGCAGCAGCTTCTTTAGCGGCGGCTTCAGCCTTGATTTCCAGCTTGGT	AEAAAKEAAAKA,VL-Cterm-Rev
oBiTE_25	GGAGGCGGAGGATCTCAGGTTCAGCTGCAGCAG	GGGGS,VH-Nterm,Fwd
oBiTE_26	GGAGGCGGAGGATCTGGTGGATCTCAGGTTCAGCTGCAGCAG	GGGGSGGS,VH-Nterm,Fwd
oBiTE_27	GCTGAAGCCGCTGCTAAAGCTCAGGTTCAGCTGCAGCAG	AEAAAKA,VH-Nterm,Fwd
oBiTE_28	GCTGAAGCCGCCGCTAAAGAAGCTGCTGCCAAAGCTCAGGTTCAGCTGCAGCAG	AEAAAKEAAAKA,VH-Nterm,Fwd
oBiTE_29	GGAGGCGGAGGATCTGACATCCAGCTGACACAG	GGGGS,VL-Nterm, Fwd
oBiTE_30	GGAGGCGGAGGATCTGGTGGATCTGACATCCAGCTGACACAG	GGGGSGGS,VL-Nterm,Fwd
oBiTE_31	GCTGAAGCCGCTGCTAAAGCTGACATCCAGCTGACACAG	AEAAAKA,VL-Nterm,Fwd
oBiTE_32	GCTGAAGCCGCCGCTAAAGAAGCTGCTGCCAAAGCTGACATCCAGCTGACACAG	AEAAAKEAAAKA,VL-Nterm,Fwd
oBiTE_33	AGATCCTCCGCCTCCAGAAGAAACGGTCACGGT	GGGGS,VH-Cterm,Rev
oBiTE_34	AGATCCACCAGATCCTCCGCCTCCAGAAGAAACGGTCACGGT	GGGGSGGS,VH-Cterm,Rev
oBiTE_35	AGCTTTAGCAGCGGCTTCAGCAGAAGAAACGGTCACGGT	AEAAAKA,VH-Cterm,Rev
oBiTE_36	AGCTTTGGCAGCAGCTTCTTTAGCGGCGGCTTCAGCAGAAGAAACGGTCACGGT	AEAAAKEAAAKA,VH-Cterm,Rev
oBiTE_37	ATCATCCTGTTTCTGGTGGCCACAGCCACAGGCGCCTATGCTCAGGTTCAGCTGCAGCAG	SS-VH_Nterm
oBiTE_38	ATCATCCTGTTTCTGGTGGCCACAGCCACAGGCGCCTATGCTGACATCCAGCTGACACAG	SS-VL-Nterm
oBiTE_39	CTTTAACAGAGAGAAGTTCGTGGCATGGTGATGGTGGTGGTGAGAAGAAACGGTCACGGT	VH_Cterm_P2A
oBiTE_40	CTTTAACAGAGAGAAGTTCGTGGCATGGTGATGGTGGTGGTGCTTGATTTCCAGCTTGGT	VL_Cterm_P2A

Supplementary Table 8: Primer sequences used for assembly of scFv sequences from CD19-NNW-scFv sublibrary, scFv sequences from CD3-scFV-sublibrary, and linker oligos
