# Supplementary material for: A high throughput bispecific antibody discovery pipeline
Source: Commun Biol. 2023 Apr 7;6:380. doi: 10.1038/s42003-023-04746-w (PMC10082157; doi:10.1038/s42003-023-04746-w)
Supplement: Supplementary file 2 — Description of Additional Supplementary Data [file 42003_2023_4746_MOESM2_ESM.docx]

**Description of Additional Supplementary Files**

**File name:** Supplementary Data 1

**Description:** The source data behind graphs in the paper.
