# Supplementary material for: A high throughput bispecific antibody discovery pipeline
Source: Commun Biol. 2023 Apr 7;6:380. doi: 10.1038/s42003-023-04746-w (PMC10082157; doi:10.1038/s42003-023-04746-w)
Supplement: Supplementary file 4 — Reporting Summary [file 42003_2023_4746_MOESM4_ESM.pdf]

nature portfolio

Corresponding author(s): Weian Zhao

Last updated by author(s): Mar 13, 2023

Reporting Summary

Nature Portfolio wishes to improve the reproducibility of the work that we publish. This form provides structure for consistency and transparency in reporting. For further information on Nature Portfolio policies, see our Editorial Policies and the Editorial Policy Checklist.

Statistics

For all statistical analyses, confirm that the following items are present in the figure legend, table legend, main text, or Methods section.

n/a	Confirmed	
☐	☒	The exact sample size (*n*) for each experimental group/condition, given as a discrete number and unit of measurement
☐	☒	A statement on whether measurements were taken from distinct samples or whether the same sample was measured repeatedly
☐	☒	The statistical test(s) used AND whether they are one- or two-sided<br>*Only common tests should be described solely by name; describe more complex techniques in the Methods section.*
☒	☐	A description of all covariates tested
☒	☐	A description of any assumptions or corrections, such as tests of normality and adjustment for multiple comparisons
☐	☒	A full description of the statistical parameters including central tendency (e.g. means) or other basic estimates (e.g. regression coefficient) AND variation (e.g. standard deviation) or associated estimates of uncertainty (e.g. confidence intervals)
☒	☐	For null hypothesis testing, the test statistic (e.g. *F*, *t*, *r*) with confidence intervals, effect sizes, degrees of freedom and *P* value noted<br>*Give P values as exact values whenever suitable.*
☒	☐	For Bayesian analysis, information on the choice of priors and Markov chain Monte Carlo settings
☒	☐	For hierarchical and complex designs, identification of the appropriate level for tests and full reporting of outcomes
☒	☐	Estimates of effect sizes (e.g. Cohen's *d*, Pearson's *r*), indicating how they were calculated

Our web collection on statistics for biologists contains articles on many of the points above.

Software and code

Policy information about availability of computer code

Data collection	LabVIEW 2017 was used for droplet sorting
Data analysis	Flow cytometry- FCS Express 7, NovoExpress Software (version 1.6.1)<br>Graphical representation- GraphPad Prism 9.4.0(673), Microsoft® Excel® for Microsoft 365 MSO (Version 2302 Build 16.0.16130.20186) 64-bit<br><br>Long read amplicon sequencing data analysis-predefined packages stringr_1.4.0 in R(version 4.0.2 (2020-06-22))<br><br>Sanger sequencing data analysis-Benchling

For manuscripts utilizing custom algorithms or software that are central to the research but not yet described in published literature, software must be made available to editors and reviewers. We strongly encourage code deposition in a community repository (e.g. GitHub). See the Nature Portfolio guidelines for submitting code & software for further information.

Data

Policy information about availability of data

All manuscripts must include a data availability statement. This statement should provide the following information, where applicable:

- Accession codes, unique identifiers, or web links for publicly available datasets
- A description of any restrictions on data availability
- For clinical datasets or third party data, please ensure that the statement adheres to our policy

The datasets generated during and/or analyzed during the current study are available from the corresponding author on reasonable request.

Human research participants

Policy information about studies involving human research participants and Sex and Gender in Research.

Reporting on sex and gender	NA
Population characteristics	NA
Recruitment	NA
Ethics oversight	NA

Note that full information on the approval of the study protocol must also be provided in the manuscript.

Field-specific reporting

Please select the one below that is the best fit for your research. If you are not sure, read the appropriate sections before making your selection.

☒ Life sciences ☐ Behavioural & social sciences ☐ Ecological, evolutionary & environmental sciences

For a reference copy of the document with all sections, see nature.com/documents/nr-reporting-summary-flat.pdf

Life sciences study design

All studies must disclose on these points even when the disclosure is negative.

Sample size	Commonly acceptable sample size of n>= 30 was typically used for quantifying reporter activation efficiency in droplets. For all other data we typically used three replicates per data point.
Data exclusions	Three clones were excluded from data analysis for Fig. 7b due to sequence ambiguity in the CDRH3 region.
Replication	Our data for analysis of diversity of integrated BiTE clones typically came from n= 1 integration experiment. We believe this is representative of our integrated BiTE library, since we do not expect integration efficiency to affect diversity. Spiking experiments (Fig. 4) were generated with over 40 data points with multiple replicates and results were consistent across replicates. Primary T cell activation and killing data in Fig. 6 were generated with n=3 replicates and results were consistent across the replicates.
Randomization	No randomization was done.
Blinding	Data collection and analysis was not performed in a blinded manner.

Reporting for specific materials, systems and methods

We require information from authors about some types of materials, experimental systems and methods used in many studies. Here, indicate whether each material, system or method listed is relevant to your study. If you are not sure if a list item applies to your research, read the appropriate section before selecting a response.

Materials & experimental systems

n/a	Involved in the study
☐	☒ Antibodies
☐	☒ Eukaryotic cell lines
☒	☐ Palaeontology and archaeology
☒	☐ Animals and other organisms
☒	☐ Clinical data
☒	☐ Dual use research of concern

Methods

n/a	Involved in the study
☒	☐ ChIP-seq
☐	☒ Flow cytometry
☒	☐ MRI-based neuroimaging

Antibodies

Antibodies used

PE-anti human CD69 antibody (#310906, Biolegend, Clone FN50), FITC-anti human CD25 antibody (#356106, Biolegend, Clone MA-251), APC anti-human CD279 antibody (#329908, Biolegend,Clone EH12.2H7), PE/Cyanine 7 anti-human CD4 antibody (#317414, Biolegend, Clone OKT4), APC/Cyanine 7 anti-human CD8 antibody (#344714, Biolegend, Clone SK1), Alexa Fluor 647 anti-human CD19 antibody (#302222, Biolegend, Clone H1B19).

Validation

Manufacturer website lists that validation of PE-anti human CD69 antibody (##310906, Biolegend) was done on Jurkat cells activated with PMA+Ionomycin. Manufacturer website also list 31 publications using this product including Kagoya Y, et al. 2018. Nat Commun. 9:1915., and Stuart T, et al. 2019. Cell. 177:1888.
Manufacturer lists validation of FITC-anti human CD25 antibody (#356106, Biolegend) done on PHA stimulated PBMCs. Manufacturer website list one reference using this product(Martinez EM, et al. 2018. SLAS Discov. 1.377083333).
Manufacturer lists validation of APC anti-human CD279 antibody (#329908, Biolegend) done on PHA stimulated PBMCs. Manufacturer website also lists 31 product citations including Renner K, et al. 2020. Cell Reports. 29(1):135-150.e9 and Zhu Y, et al. 2019. Cell Stem Cell. 25:542.
Manufacturer lists validation of PE/Cyanine 7 anti-human CD4 antibody (#317414, Biolegend) on human peripheral blood lymphocytes. Manufacturer website also lists 19 product citations including Delacher M, et al. 2021. Immunity. 54(4):702-720.e17 and Keskin DB, et al. 2019. Nature. 565:234.
Manufacturer lists validation of APC/Cyanine 7 anti-human CD8 antibody (#344714, Biolegend) on human peripheral blood lymphocytes. Manufacturer website also lists 26 product citations including Goenka A, et al. 2021. Cell Reports Medicine. :100327 and Graham C, et al. 2021. Immunity. 54(6):1276-1289.e6
Manufacturer lists validation of Alexa Fluor 647 anti-human CD19 antibody (#302222, Biolegend) on human peripheral blood lymphocytes. Manufacturer website lists 4 product citations including Nerreter T, et al. 2019. Nat Commun. 10:3137 and Manian KV et al. 2018. Stem cell research. 29:148-151.

Eukaryotic cell lines

Policy information about cell lines and Sex and Gender in Research

Cell line source(s)

Raji(ATCC CCL-86,CD19+ cell lines) were used as CD19+ cells in droplet functional screening assays and in-vitro functional tests. HEK293_LP(#AST-1305, Applied Stemcell, Single copy landing pad integrated cell line) were used for integration of BiTE variant library and Blinatumomab, Jurkat E6.1 (ATCC TIB-152, T lymphoblast cell line) were used as CD19- control cell lines. NALM-6(ATCC CRL-3273) and SU-DHL-6(CRL-2959) were included CD19+ positive cell lines for comparing surface expression of CD19 on engineered HEL293_LP cells. Jurkat-ZsG and Jurkat-E2C were engineered in-house from Jurkat E6.1 cell lines.

Authentication

Cell lines were obtained and authenticated by credible vendors (e.g.ATCC) and we did not perform additional authentication.

Mycoplasma contamination

All cell lines when we obtained from the vendors were negative for mycoplasma contamination. We did not perform further mycoplasma tests.

Commonly misidentified lines
(See ICLAC register)

No cell lines were misidentified.

Flow Cytometry

Plots

Confirm that:

☒ The axis labels state the marker and fluorochrome used (e.g. CD4-FITC).

☒ The axis scales are clearly visible. Include numbers along axes only for bottom left plot of group (a 'group' is an analysis of identical markers).

☒ All plots are contour plots with outliers or pseudocolor plots.

☒ A numerical value for number of cells or percentage (with statistics) is provided.

Methodology

Sample preparation	Cell cultures were detached, by trypsinization (if necessary), washed with staining buffer(PBS+2%FBS) and resuspended in staining buffer containing antibody /antibody cocktail at appropriate concentrations. After staining for 20 mins at room temperature, the cells were centrifuged and resuspended in PBS+2%FBS and used for flow cytometry.
Instrument	NovoCyte Penteon U7V7B6Y6R4 Flow Cytometer System
Software	NovoExpress software, FCS Express 7 Research (7.06.0015)
Cell population abundance	No flow cytometric sorting was done. All sorting was done on the single cell droplet microfluidic platform.
Gating strategy	For Fig. 5d, we first applied forward /side scatter parameters to exclude cell debri (FSC/SSC). We then used FSC-A(FSC-area) and FSC-H (FSC-height) to gate for single cells. Jurkat-ZsG and Raji cells were separated by gating based on basal GFP fluorescence of Jurkat-ZsG. The increased GFP intensity of Jurkat-ZsG cells was then gated based on unactivated Jurakt-ZsG cells.<br>Gating strategy for flow cytometric analysis of in vitro activation efficiencies (Fig. 6(a-d)) followed a similar scheme. After removing cell debri (FSC/SSC) and gating singlets(FSC-H/FSC-A), we gated for 7AAD- cells (live cells) and then sub-gated for CD4+ and CD8+ populations. The CD4+ and CD8+ subpopulations were gated for dual positive CD69+CD25+ cells and PD-1+.<br>For Raji cell apoptosis (Fig. 6e), after removing cell debri (FSC/SSC) and isolating singlets(FSC-H/FSC-A), we gated out T cells by gating out CD4+CD8+ population. Percentage of Raji cells that were Annexin V+ and 7AAD+ were then obtained from the non-T cell population.

☒ Tick this box to confirm that a figure exemplifying the gating strategy is provided in the Supplementary Information.
